# Antibiotic resistance profile of common uropathogens during COVID-19 pandemic: hospital based epidemiologic study

**DOI:** 10.1186/s12866-023-02773-5

**Published:** 2023-01-25

**Authors:** Ahmed M. Abdel Gawad, Walaa Mohamed Omar Ashry, Sherief El-Ghannam, Mahmoud Hussein, Ahmed Yousef

**Affiliations:** 1 Department of Urology, Damietta Faculty of Medicine, Al-Azhar University, New Damietta City, Egypt; 2 Department of Medical Microbiology and Immunology, Damietta Faculty of Medicine (Girls), Al-Azhar University, New Damietta City, Egypt; 3 Department of Clinical Pathology, Damietta Faculty of Medicine, Al-Azhar University, New Damietta City, Egypt; 4 Department of Forensic Medicine and Clinical Toxicology, Damietta Faculty of Medicine, Al-Azhar University, New Damietta City, Egypt; 5 Department of Public Health and Community Medicine, Damietta Faculty of Medicine, Al-Azhar University, New Damietta City, Egypt

**Keywords:** Antimicrobial resistance, Antimicrobial stewardship, COVID-19, Uropathogens, Urinary tract infection

## Abstract

**Background:**

Antimicrobial resistance has a direct impact on the ability to treat common infections, and this was worsened during the COVID-19 pandemic. Worldwide surveillance studies are lacking and resistance rates vary spatially, so frequent local surveillance reports are required to guide antimicrobial stewardship efforts.

This study aims to report our common local uropathogens and their antibiogram profiles in our community during the COVID era.

**Methods:**

A retrospective study included patients referred to our urology units with urine culture and sensitivity. All bacterial strains were identified, and their antibiotic susceptibilities were tested.

**Results:**

Out of 2581 urine culture results recruited, 30% showed microbiological proof of infection. The majority, 486 (63.4%), were isolated from females. The most frequent isolates were *Escherichia coli* (44.4%) and *Staphylococcus aureus* (17.8%). The resistance rates ranged from 26.9 to 79.7%. Piperacillin-tazobactam antibiotic had the lowest resistance rate. The multi-drug resistance pattern was recorded in 181 (23.9%) of the isolates; 159/597 (26.6%) Gram-negative and 22/160 (13.8%) Gram-positive isolates.

**Conclusions:**

Alarming rates of antimicrobial resistance were detected, which stresses the significance of following infection control policies and establishing national antimicrobial stewardship standards.

## Introduction

Worldwide, 150–250 million urinary tract infections (UTIs) are diagnosed annually in outpatient settings and long-term care facilities. UTIs are a source of illness in infant boys, older females, and men of all ages, with societal consequences including health care expenditures and period missed from workplace. Repeated recurrences, pyelonephritis, and cystitis in young children, pre-term delivery, and problems related to frequent antimicrobial usage, such as high-level antibiotic resistance are all serious consequences [1].

Uropathogenic bacteria are the main cause of UTIs. Other species that can cause UTIs include fungus, viruses, and parasites [2]. The clinical medical history and physical examination are important in determining UTI. However, the standard culture-based technique (uropathogen is cultivated, identified, and tested for antibiotic sensitivity) is the standard for diagnosis and treatment [3]. This has an average delay of 2-3 days, making it ineffective for patients with complicated UTIs who are at risk of developing life-threatening urosepsis. In such cases, physicians are required to start empiric antibiotic therapy to manage the infection. If the chosen antibiotic regimen is insufficient in severely ill patients with a low margin for error, knowledge of the common local pathogens and their local antibiogram profile dictates the optimal choice of empiric therapy [4].

Awareness of local and regional antimicrobial susceptibility variations is one method for improving antibiotic prescription and, at most in part, preventing the spread of antimicrobial resistance (AMR) [5].

AMR surveillance studies are insufficient, and resistance rates vary widely, so it is essential to develop periodic local surveillance reports to guide empiric antibiotic therapy until patient-specific data becomes available [6].

Antibiotic usage has increased since the emergence of the COVID-19 pandemic. Antibiotic misuse has led to the emergence of antibiotic resistant organisms. Resistant pathogens thrive in healthcare facilities, placing all patients at risk, regardless of their medical conditions [7].

This work aims to describe the common local uropathogens and their antibiogram profiles in the Department of Urology, Damietta Faculty of Medicine, Al-Azhar University, Egypt, during the COVID-19 pandemic, over the year 2021.

## Materials and methods

### Study design

A retrospective study was conducted in the Urology Department, Damietta Faculty of Medicine, Al-Azhar University, Egypt, to report on uropathogenic bacteria associated with UTIs and their antibiotic resistance patterns from confirmed cases. Primary data was gathered (from January 1st 2021 till the end of December 2021). Participants of all ages who presented to outpatient and inpatient units with urine culture and sensitivity were included in the study population. Participants in the inpatient unit with an indwelling urinary catheter or had a urinary tract infection after 48 hours of admission were excluded.

### Collection of specimens

Two thousand five hundred eighty-one clean-catch midstream urine samples were collected for both culture and sensitivity. Adult patients collected midstream urine specimens in sterile cups, whereas nurses collected specimens from newborn and infant patients into sterile urine bags. Colony counts of more than 10^5^ CFU/mL were considered significant [4].

### Pathogen isolation

Ten ml of midstream urine samples were centrifuged for 10 minutes at 2000 rpm. To obtain pure, isolated colonies, 0.5 ml of urine sediments were suspended and cultured with a calibrated loop on various selective and differentiating media (such as CLED agar, MacConkey agar, blood agar, chocolate agar, and sabouraud agar); plates were incubated aerobically at 37 °C for 24–48 h. Subculturing of mixed growth cultures was done to ensure pure cultures.

### Pathogens identification

#### Observation of the cultures

All the incubated plates were observed for the colony morphology, size, color, swarming, and hemolytic action on blood agar.

#### Microscopic examination

Gram staining was performed on different colonies and examined for their shape, arrangement, and Gram reaction.

#### Biochemical examination

Selected colonies were subjected to further biochemical examinations such as carbohydrate utilization tests, triple sugar iron test, catalase test, coagulase test, oxidase test, indole production test, methyl red test, Voges-Proskauer test, urease test, citrate utilization test, bile aesculin test, and motility test.

The obtained data were collected, analyzed and interpreted following the Clinical Laboratory Standards Institute (CLSI) [8].

### Antibiotic sensitivity testing

Antibiotic susceptibility testing was carried out on clinically significant bacterial isolates using the Kirby Bauer disc diffusion method on Mueller-Hinton agar following CLSI recommendations. The antibiotic discs and concentrations (μg) used were as follows: amikacin (30), amoxicillin-clavulanic acid (30), ceftazidime (30), ciprofloxacin (5), TMP/SMX (25), gentamicin (10), imipenem (10), meropenem (10), nitrofurantoin (30), piperacillin-tazobactam (40), ceftriaxone (30), fusidic acid (5), vancomycin (30), cefoxitin (30), cefuroxime (30), cefepime (30), levofloxacin (5), erythromycin (15), tetracycline (30), clindamycin (10), teicoplanin (30). The plates were incubated at 37 °C for 24 hours. The diameters of the zones of inhibition of antibiotics were measured and interpreted using the CLSI guidelines [8].

### Data analysis

Data was collected in Excel form, checked, entered, and analyzed using SPSS version 23 for data processing and statistics. Categorical data were presented as numbers and percentages. To assess the normality of quantitative data, the Kolmogorov-Smirnoff test was applied. Quantitative data were expressed as mean ± standard deviation for normal distribution.

## Results

### Study subjects

Two thousand five hundred eighty-one urine culture results from both outpatient and inpatient units were recorded. Microbiological proof of infection was found in 766 (30%) of the samples.

The prevalence of UTIs in patients under the age of 13 years was 375 (49%), while the prevalence in patients over the age of 18 years was 361 (47%), and only 30 (4%) were between the ages of 13 and 18 years (Table 1).

**Table 1:** Demographic characteristics of culture positive patients

	Total (*n* = 766 - 100.0%)	
	Frequency N	%
**Age**		
< 13 years old	375	49
13 - 18 years old	30	4
> 18 years old	361	47
**Sex**		
Male	280	36.6.0
Female	486	63.4

Most of the uropathogens were retrieved from female patients 486 (63.4%), whereas males constituted only 280 (36.6%) patients. The male to female ratio was 0.58: 1 (Table 1).

In regards of related comorbidity, about 56 patients had hypertension, 45 had diabetes mellitus, and 19 had chronic renal disease (Table 2).

**Table 2:** Medical history and co-morbidity

Morbidity	Frequency	%
Hypertension	56	40.6
Diabetes mellitus	45	32.6
Chronic kidney diseases	19	13.8
HIV infection	9	6.5
Others (Bronchial asthma &CVS	9	6.5
Total	138	100.0

### Uropathogenic bacterial species

While 160/766 (20.9%) were Gram-positive organisms, the majority of the isolates 591/766 (77.1%) were Gram-negative organisms, and only six (0.8%) were mixed (Table 3).

**Table 3:** Types and frequency of bacterial species isolated from UTI patients’ urine cultures according to age and sex

Uropathogens Total (766)			Frequency among patients									
			Age						Sex			
			< 13 years old ***n*** = 375		13 - 18 years old ***n*** = 30		> 18 years old ***n*** = 361		Male ***n*** = 280		Female ***n*** = 486	
	N	%	N	%	N	%	N	%	N	%	N	%
***E. coli***	340	44.4	144	38.4	11	36.7	185	51.2	107	38.2	233	48.0
***Klebsiella***	106	13.8	57	15.2	1	3.3	48	13.3	43	15.4	63	13
***Enterobacter***	60	7.8	25	6.7	3	10.0	32	8.9	19	6.8	41	8.4
***Pseudomonas***	35	4.6	18	4.8	0	0.0	17	4.7	20	7.1	15	3.1
***Proteus***	48	6.3	36	9.6	4	13.3	8	2.2	20	7.1	28	5.8
***Acinetobacter***	1	0.1	0	0.0	0	0.0	1	0.3	1	0.4	0	0.0
***Serratia***	1	0.1	1	0.3	0	0.0	0	0.0	1	0.4	0	0.0
***S. aureus***	136	17.8	78	20.8	10	33.3	48	13.3	54	19.3	82	16.9
***Enterococcus***	24	3.1	11	2.9	0	0.0	13	3.6	12	4.3	12	2.5
***C. albicans***	9	1.2	3	0.8	1	3.3	5	1.4	3	1.1	6	1.2
***Mixed***	6	0.8	2	0.5	0	0.0	4	1.1	0	0.0	6	1.2

Ten different species of uropathogens were isolated. They are listed in order of frequency, from most to least frequent, *E. coli* 340 (44.4%) > *S. aureus* 136 (17.8%) > *Klebsiella* spp. 106 (13.8%) > *Enterobacter* 60 (7.8%) > *Proteus* spp. 48 (6.3%) > *Pseudomonas* 35 (4.6%) > *Enterococcus* spp. 24 (3.1%) > *Candida albicans* 9 (1.2%) > *Serratia* 1 (0.1%) and *Acinetobacter* 1 (0.1%) (Table 3).

Data on the prevalence of uropathogens by age and gender found that *E. coli* was the most frequently identified species among participants over 18 years. The distribution of *E. coli* and *S. aureus* was almost the same among both those under the age of 13 years and those between 13 and 18 years (Table 3). Females were more prone to infection with *E. coli*, whereas males were more likely to be infected with *S. aureus*, *Klebsiella* spp., *Pseudomonas*, and *Proteus* spp. (Table 3).

Mixed infection with more than one type of organism was reported in six patients; all were female, two of whom were under the age of 13 years, and four were over 18 years (Table 3).

### Antibiotic susceptibility

Twenty-one antibiotics were tested for susceptibility profiles. The overall resistance rates are shown in Table 4. The antibiotic that exhibited the highest level of susceptibility and the lowest resistance rate was piperacillin-tazobactam (> 73.1%), followed by cefoxitin (71.2%), cefepime (68.6%), meropenem (63.9%), and ceftriaxone (63.5%). On the other hand, the antibiotics that exhibited the highest level of resistance were erythromycin (> 79.7%), gentamicin (76.9%), teicoplanin (75%), and TMP/SMX (71.6%).

**Table 4:** Susceptibility profile of tested antibiotics

	Total	Sensitive		Resistant	
	N	N	%	N	%
**Meropenem**	545	348	63.9	197	36.1
**Imipenem**	414	149	36	265	64
**Piperacillin-Tazobactam**	510	373	73.1	137	26.9
**Amoxicillin Clavulanic Acid**	539	177	32.8	362	67.2
**Ceftriaxone**	167	106	63.5	61	36.5
**Cefepime**	118	81	68.6	37	31.4
**Ceftazidime**	378	155	41.0	223	59.0
**Cefuroxime**	464	221	47.6	243	52.4
**Cefoxitin**	177	126	71.2	51	28.8
**Amikacin**	561	279	49.7	282	50.3
**Gentamicin**	668	154	23.1	514	76.9
**Ciprofloxacin**	696	359	51.6	337	48.4
**Levofloxacin**	456	271	59.4	185	40.6
**TMP/SMX** ^a^	599	170	28.4	429	71.6
**Nitrofurantoin**	565	236	41.8	329	58.2
**Vancomycin**	148	84	56.8	64	43.2
**Erythromycin**	143	29	20.3	114	79.7
**Tetracycline**	154	49	31.8	105	68.2
**Clindamycin**	149	66	44.3	83	55.7
**Fusidic Acid**	144	81	56.3	63	43.8
**Teicoplanin**	20	5	25.0	15	75.0

^a^ *TMP/SMX* Trimethoprim-Sulfamethoxazole

The antibiogram resistance profile of different Gram-negative isolates is reported in Table 5. *E. coli* demonstrated high resistance to gentamicin (82.3%), amoxicillin-clavulanic acid (69.2%), TMP/SMX (68.2%), imipenem (56.3%), and ceftazidime (54.6%). On the other hand, it was more susceptible to piperacillin-tazobactam (72.2%) and cefepime (71.2%).

**Table 5:** Antibiogram resistance profile of Gram-negative and Gram- positive isolates

Antibiotics	Gram-negative								Gram-Positive	
	***E. coli*** (***N*** = 340)	***Klebsiella*** (***N*** = 106)	***Enterobacter*** spp. (***N*** = 60)	***Pseudomonas*** spp. (***N*** = 35)	***Proteus*** spp. (***N*** = 48)	***Actinobacter*** spp. (***N*** = 1)	***Serratia*** spp. (***N*** = 1)	***Mixed*** (***N*** = 6)	***S. aureus*** (***N*** = 136)	***Enterococcus*** spp. (***N*** = 24)
	n (%)	n (%)	n (%)	n (%)	n (%)	n (%)	n (%)	n (%)	n (%)	n (%)
**Meropenem**	134 (41.6)	30 (30)	8 (15)	5 (38)	13 (30)	NT	1 (100)	4 (67)	NT	2 (66.7)
**Imipenem**	111 (56.3)	61 (69)	36 (67)	25 (81)	28 (78)	NT	NT	3 (60)	NT	1 (50)
**Piperacillin-Tazobactam**	81 (27.8)	26 (28)	15 (38)	7 (22)	5 (12)	NT	1 (100)	1 (17)	NT	1 (50)
**Amoxicillin Clavulanic Acid**	218 (69.2)	69 (70)	32 (58)	11 (79)	24 (52)	NT	1 (100)	1 (100)	NT	1 (50)
**Ceftriaxone**	31 (31.6)	11 (39)	6 (36)	6 (60)	6 (50)	NT	NT	1 (50)	NT	NT
**Cefepime**	21 (28.8)	7 (39)	6 (40)	3 (60)	NT	NT	NT	NT	NT	NT
**Ceftazidime**	95 (54.6)	59 (66)	31 (61)	7 (54)	21 (55)	NT	NT	2 (67)	6 (86)	2 (100)
**Cefuroxime**	120 (50.8)	56 (45)	26 (47)	12 (86)	22 (49)	NT	1 (100)	3 (50)	2 (100)	1 (50)
**Cefoxitin**	18 (26.5)	2 (12)	5 (21)	3 (75)	6 (75)	1 (100)	NT	NT	16 (31)	NT
**Amikacin**	171 (54.6)	38 (39)	25 (45)	14 (45)	25 (60)	1 (100)	NT	2 (33)	5 (42)	1 (50)
**Gentamicin**	247 (82.3)	76 (79)	41 (75)	21 (60)	33 (77)	1 (100)	NT	5 (83)	82 (66)	8 (100)
**Ciprofloxacin**	153 (50)	41 (41)	26 (46)	13 (41)	10 (22)	1 (100)	1 (100)	3 (50)	74 (59)	15 (65.2)
**Levofloxacin**	83 (39.7)	16 (36)	15 (42)	4 (40)	1 (5)	NT	NT	NT	56 (49)	10 (52.6)
**Tmp/Smx** ^a^	212 (68.2)	70 (70)	46 (82)	9 (75)	27 (61)	1 (100)	1 (100)	4 (67)	54 (87)	5 (83.3)
**Nitrofurantoin**	136 (47.1)	76 (75)	30 (67)	12 (86)	41 (91)	NT	1 (100)	5 (83)	17 (38)	11 (61.1)
**Vancomycin**	1 (100)	NT	NT	2 (100)	NT	1 (100)	NT	NT	53 (43)	7 (35)
**Erythromycin**	1 (100)	NT	NT	1 (100)	NT	NT	NT	NT	107 (80)	5 (71.4)
**Tetracycline**	2 (100)	NT	NT	1 (50)	NT	NT	NT	2 (100)	87 (69)	13 (72.2)
**Clindamycin**	1 (100)	NT	NT	12 (92)	NT	1 (100)	NT	NT	63 (49)	6 (100)
**Fusidic Acid**	5 (100)	NT	NT	1 (100)	1 (100)	1 (100)	NT	NT	53 (41)	2 (28.6)
**Teicoplanin**	NT	NT	NT	1 (100)	NT	NT	NT	1 (1)	2 (50)	11 (84.6)

*N* Number of Isolates, *n* Resistance Number, % Resistance Percentage, *NT* Not Tested

^a^ *TMP/SMX* Trimethoprim-Sulfamethoxazole

*Klebsiella* spp. isolates were more resistant to gentamicin (79%), nitrofurantoin (75%), TMP/SMX (70%), and amoxicillin-clavulanic acid (70%) but less resistant to cefoxitin (12%). *Enterobacter* spp. isolates were more resistant to TMP/SMX (82%) and gentamicin (75%). *Pseudomonas* isolates were resistant to clindamycin (92%), cefuroxime and nitrofurantoin (86%), and imipenem (81%). A few Pseudomonas isolates were shown to be completely resistant (100%) to vancomycin, erythromycin, fusidic acid, and teicoplanin.

*Proteus* isolates exhibited strong resistance to nitrofurantoin (91%) and low resistance to levofloxacin (5%). *Actinobacter* spp. and *Serratia* spp. had one identified strain, and this isolate exhibited extremely high resistance levels (100%) to around seven antibiotics. In mixed infections, isolates exhibited 100% resistance to amoxicillin-clavulanic acid and tetracycline.

The antibiogram resistance profile of Gram -positive bacteria is summarized in Table 5. *S. aureus* isolates were resistant to ceftazidime (86%) and TMP/SMX (87%). Only 2 *S. aureus* isolates showed complete resistance to cefuroxime (100%).

Teicoplanin resistance was observed in *Enterococcus* spp. isolates (84.6%). *Enterococcus* spp. showed complete resistance to gentamicin, clindamycin, and ceftazidime.

Multi-drug resistance (MDR) pathogens are those resistant to one or more antibiotics from two or more antibiotic categories [9]. The incidence of MDR in various isolates is seen in Table 6. A MDR pattern was recorded in 181 (23.9%) of the total bacterial isolates (*n* = 757), of which 159/597 (26.6%) in Gram-negative and 22/160 (13.8%) in Gram-positive bacterial isolates showed resistance to two or more classes of antimicrobial agent (Table 6).

**Table 6:** Multi-drug resistance pattern of bacterial isolates

	MDR N (%)		N (%)
**Overall Gram-negative**	159 (26.6)	***E. coli***	95 (27.9)
		***Klebsiella*** **spp**.	30 (28.3)
		***Enterobacter*** **spp.**	15 (25)
		***Pseudomonas*** **spp.**	7 (20)
		***Proteus*** **spp.**	10 (20.8)
		***Acinetobacter*** **spp.**	0
		***Serratia*** **spp.**	0
		***Mixed***	2 (33.3)
**Overall Gram-positive**	22 (13.8)	***S. aureus***	17 (12.5)
		***Enterococcus*** **Spp.**	5 (20.8)

## Discussion

Complicated UTIs are a major health concern that necessitates the detection of uropathogens and their susceptibility patterns to avoid antibiotic misuse. Local surveillance for bacterial resistance is critical for creating effective antimicrobial stewardship guidelines [10].

According to new research, the COVID-19 pandemic has altered the resistance patterns of various bacteria due to antibiotic misuse for the treatment of viral illness. Awareness of the resistance patterns of the most common uropathogens is essential for providing quality of practice in overcoming these infections [11].

The current study mainly utilized clinical data from our filing system to show the frequency of uropathogens associated with UTIs and their antibiotic response profiles to routinely used antibiotics in the Urology department at Al-Azhar University Hospital in Damietta, Egypt, between January 2021 and December 2021.

In the present study, the microbiological identification of infection was diagnosed in 30% (766/2581) of the urine culture samples. The current findings are supported by the conclusions of many previous studies in Nigeria [12] and Central Europe [5]. In contrast, some studies have reported higher prevalence rates of 45% [13] and 70.83% [14]. This discrepancy may be explained by variation in research population characteristics, environmental circumstances, and techniques.

As in prior research, the majority of UTI patients (63.4%) were females. This is to be expected, and it is similar to the findings of other research [3, 15]. This is explained by the difference in the anatomy of the female urinary tract compared to the male, which allows bacteria to easily access the bladder from the urethral meatus and perineum [16].

UTIs etiological agents and antibiotic susceptibility or resistance patterns vary by geographical region, age, and sex [17]. As expected, 99% of UTIs were of bacterial origin, with Gram-negative bacteria being the cause in 591 (77.1%), similar to prior published data with somewhat varying percentages [12].

In this study, frequent pathogens were isolated. *E. coli* was the most common pathogen, representing 340 (44.4%) of the uropathogen, followed by *S. aureus* 136 (17.8%) and *Klebsiella* spp. 106 (13.8%). These findings were consistent with previous reports both in Egypt [13] and other different nations, e.g. Germany [18].

The current study confirmed earlier research that females were infected with *E. coli* at a greater incidence (48.0%) than males (38.2%) [19]. Only *Pseudomonas* was more prevalent in males than in females. This is in line with that reported by Mirsoleymani et al. [20].

Resistance among bacterial uropathogens to routinely used antibiotics has developed, leaving clinicians with limited alternatives for UTIs treatments. Due to the lack of novel antibiotics, infections generated by antimicrobial-resistant bacteria are related to increased treatment failure rates, increased hospitalizations, higher costs, and death [6].

In this study, among the tested antibiotics, the lowest resistance rate was 26.9% for piperacillin-tazobactam, followed by cefoxitin (28.8%), cefepime (31.4%), meropenem (36.1%), and ceftriaxone (36.5%). Given that for empiric therapy of severe infections, resistance rates should not exceed 10% [21], and our lowest resistance rate was 26.9%, we have few alternatives in urosepsis. However, these antibiotics should be considered, alone or in combination, for the initial empiric management of severe UTIs. On the other hand, the highest levels of resistance were recorded for erythromycin (79.7%), gentamicin (76.9%), teicoplanin (75%), and TMP/SMX (71.6%). As a result, these antibiotics are not recommended to be used as empirical treatment for UTIs [21]. The levels of resistance and sensitivity of different antibiotics varied between studies, but the current findings were closely related to those observed by Mirsoleymani et al. [20].

The resistance rate for carbapenems tested in our study was 36.1% for meropenem and 64% for imipenem. Such high rates of resistance are in agreement with previous studies in Egypt [22]. It has been attributed to the production of carbapenemase genes, of which bla_NDM_ and bla_OXA_ genes are predominant in the Middle East and Egypt [23].

For aminoglycosides, gentamicin resistance varied by country, with Turkey having the highest rate of resistance (94.5%) [24]. In comparison, India has substantially lower rates of resistance (32.6%) [25]. In our work, gentamicin had the second highest rate of resistance among tested antibiotics (76.9%), consistent with this report from Egypt [26]. For amikacin, our rate of resistance (50.3%) was followed by previous reports [24].

Resistance rates for quinolones were (48.4%) and (40.6%) for ciprofloxacin and levofloxacin, respectively. These rates were consistent with those reported by Labah et al. [14] but were lower than those reported by the Abdelkhalik group [26].

Also, for Beta-lactam antibiotics, the current study showed resistance rates of 67.2, 59, and 52.4% for amoxicillin-clavulanic acid, ceftazidime, and cefuroxime, respectively, which was in agreement with previous reports [14, 26].

Finally, resistance rates for narrow-spectrum antibiotics (TMP-SMX and nitrofurantoin), which are frequently used for uncomplicated UTIs, were 71.6 and 58.2%, respectively. These rates agreed with those reported by Labah et al. [14]. Controversially, Randrianirina et al. [27] noted higher sensitivity to TMP-SMX. These differences in the results can be attributed to the emergence of resistant strains due to their recurrent misuse.

MDR is defined as pathogens resistant to one or more antibiotics in two or more classes of antibiotics [9]. Infections caused by MDR organisms have extremely limited therapeutic options. In our work, MDR was more prevalent among Gram-negative bacteria (26.6%) compared to 13.8% in Gram-positive ones. Labah et al. recorded MDR (58.26%) in Gram-negative bacteria [14].

The higher rates of resistance to tested antibiotics in our study are of great concern. They may be the result of inadequate infection control strategies and the misuse of these life-saving drugs. Antibiotics were administered to 49.8% of patients treated in outpatient healthcare institutions, and antibiotics were available without a prescription [22].

Furthermore, expanding evidence clearly indicates the transmission of resistance, especially through chicken meat, which has the highest levels of contamination by resistant germs since antimicrobials are frequently used in veterinary care for infection prevention and treatment, and antimicrobial-resistant bacteria have been detected in veterinary isolates in Egypt [28].

### Limitations of this study

- Because this study was focused on a single health institution, it may not accurately reflect the overall condition of the community.
- Due to restricted laboratory resources, anaerobic bacteria, fungi, and viral agents that cause UTIs were not examined.
- Because sensitivity rates differ among healthcare institutions, the results may not be predictive and replicable in other healthcare institutions.
- There might be some observational errors, particularly when assessing the antibacterial inhibition zone.

Regarding these limits, the study gives sufficient updated information on UTIs, antimicrobial susceptibility profiles, and related variables.

## Conclusion

Our study focused on the cumulative 2021 antibiogram results of our institution. Alarming rates of AMR were detected, which focus on the importance of monitoring the resistance patterns, respecting infection control measures, and implementing antimicrobial stewardship protocols in an attempt to preserve some of these life-saving drugs for future generations. The results of our work will be tailored for our inpatient and outpatient units, plus other units of our institution.

## Data Availability

The datasets used and/or analyzed during the current study available from the corresponding author on reasonable request.

## Abbreviations

AMR: Antimicrobial Resistance
CLSI: Clinical and Laboratory Standards Institute
MDR: Multi-Drug Resistance
TMP/SMX: Trimethoprim-Sulphamethoxazole
UTIs: Urinary Tract Infections
